# Development and evaluation of an open source software tool for deidentification of pathology reports

**DOI:** 10.1186/1472-6947-6-12

**Published:** 2006-03-06

**Authors:** Bruce A Beckwith, Rajeshwarri Mahaadevan, Ulysses J Balis, Frank Kuo

**Affiliations:** 1 Department of Pathology, Beth Israel Deaconess Medical Center, 330 Brookline Ave., Boston, MA, USA; 2 Department of Pathology, Harvard Medical School, 25 Shattuck Street, Boston, MA, USA; 3 Department of Pathology, Massachusetts General Hospital, 55 Fruit Street, Boston, MA, USA; 4 Department of Pathology, Brigham & Women's Hospital, 75 Francis Street Boston, MA, USA

## Abstract

**Background:**

Electronic medical records, including pathology reports, are often used for research purposes. Currently, there are few programs freely available to remove identifiers while leaving the remainder of the pathology report text intact. Our goal was to produce an open source, Health Insurance Portability and Accountability Act (HIPAA) compliant, deidentification tool tailored for pathology reports. We designed a three-step process for removing potential identifiers. The first step is to look for identifiers known to be associated with the patient, such as name, medical record number, pathology accession number, etc. Next, a series of pattern matches look for predictable patterns likely to represent identifying data; such as dates, accession numbers and addresses as well as patient, institution and physician names. Finally, individual words are compared with a database of proper names and geographic locations. Pathology reports from three institutions were used to design and test the algorithms. The software was improved iteratively on training sets until it exhibited good performance. 1800 new pathology reports were then processed. Each report was reviewed manually before and after deidentification to catalog all identifiers and note those that were not removed.

**Results:**

1254 (69.7 %) of 1800 pathology reports contained identifiers in the body of the report. 3439 (98.3%) of 3499 unique identifiers in the test set were removed. Only 19 HIPAA-specified identifiers (mainly consult accession numbers and misspelled names) were missed. Of 41 non-HIPAA identifiers missed, the majority were partial institutional addresses and ages. Outside consultation case reports typically contain numerous identifiers and were the most challenging to deidentify comprehensively. There was variation in performance among reports from the three institutions, highlighting the need for site-specific customization, which is easily accomplished with our tool.

**Conclusion:**

We have demonstrated that it is possible to create an open-source deidentification program which performs well on free-text pathology reports.

## Background

The value of studying information contained within the medical record of patients has long been recognized. One of the issues related to using such medical information for research purposes has been protecting patient privacy. Currently, investigators wishing to use medical records for research purposes have three options: obtain permission from the patients, obtain a waiver of informed consent from their Institutional Review Board or use a data set that has had all (de-identified data set) or most (limited data set) of the identifiers removed [1,2]. The Health Insurance Portability and Accountability Act [2] (HIPAA) specifies that a de-identified data set can be created by removal of nineteen specific types of identifiers constitutes deidentification of the medical records (see Table 1). These identifiers include names, ages, dates, addresses, and identifying codes of patients, their relatives, household members and employers.

**Table 1:** Identifiers that must be removed to deidentify medical data per HIPAA.

**Identifier Type**
Names
Geographic subdivisions smaller than a State *
All elements of dates (except year)
All ages over 89 *
Telephone numbers
Fax numbers
Electronic mail addresses
Social security numbers
Medical record numbers
Health plan beneficiary numbers
Account numbers
Certificate/license numbers
Vehicle identifiers and serial numbers, including license plate numbers
Device identifiers and serial numbers
Web Universal Resource Locators (URLs)
Internet Protocol (IP) address numbers
Biometric identifiers, including finger and voice prints
Full face photographic images and any comparable images
Any other unique identifying number, characteristic, or code

* Additional details are given in the regulation text.

Note that these categories refer only to identifiers that concern an individual, their relatives, employers or household members

Each year, pathologists in the United States examine millions of tissue samples. This results in the creation and storage of vast numbers of paraffin embedded tissue samples. These specimens have been examined and characterized by a pathologist and a large proportion of recent samples have reports available in electronic format. In recognition of this situation, the National Cancer Institute proposed the development of the "Shared Pathology Informatics Network" (SPIN). The SPIN consortium has successfully demonstrated a prototype of a web-based, searchable, peer-to-peer network for identifying and locating pathologic tissue samples at various institutions throughout the country by searching information contained within pathology reports [3]. Since the ultimate goal of this network is to provide researchers throughout the country access to tissue specimens, it is absolutely necessary to deidentify the contents of the surgical pathology reports that form the core of the information that is contained within the network.

To date, a number of automated text deidentifiers (scrubbers) have been described [4-11]. We are aware of four reports of systems, which have been designed for, or tested upon, pathology reports [4-7]. However, one of the systems is proprietary [4], the second was not available when we started this project [5], the third was designed only for removing proper names [6], and the final system works in part by altering the contents of the text [7]. Therefore, we undertook to develop an open source text scrubber optimized for surgical pathology reports.

## Implementation

The software was developed using the following open source tools: Java (Sun Microsystems, San Jose, CA) [12], MySQL (Uppsala, Sweden) [13], and JDOM (JDOM project) [14]. Our source code [see Additional file 2] is freely available at the SPIN website [15], under the GNU General Public License terms [16].

**Figure 1:**
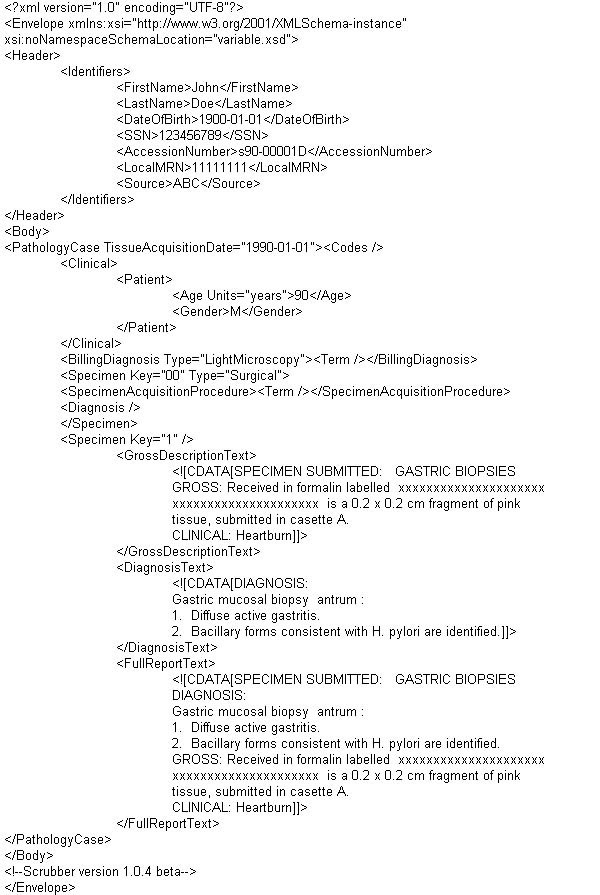
**Example surgical pathology report in SPIN XML format**. This is an example of a simple (and fictitious) surgical pathology report, which has been converted into the SPIN XML schema and deidentified by our scrubber. The schema supports a great amount of detail, but only a few of the elements are mandatory. This example shows the minimal elements needed to have a valid XML file which the scrubber will process. The format is somewhat redundant as the textual portions of the report are included twice, once in the <GrossDescriptionText> and <DiagnosisText> elements and once in the combined <FullReportText> element. Note that the original report contained the phrase 'Received in formalin labelled "John Doe"...'. The scrubber replaced "John Doe" with "xxxxxxxxxxxxxxxxxxxxx xxxxxxxxxxxxxxxxxxxxx".

As part of the SPIN project, we defined an XML schema (available at the SPIN website), which can accommodate the various types of information contained within a pathology report. This format includes a header portion that contains demographic information about the patient from which the pathology specimen was obtained, such as name, medical record number, date of birth and social security number. In addition, it includes information about the pathology report, such as the accession number and the pathology department that generated the report. The advantage of using this standardized format is that reports from a variety of sources can be transformed into this common format and then a single scrubber (and other tools such as an automated UMLS coder) can easily process reports from many different source institutions. An example of a simple surgical pathology report in the minimal SPIN XML format is shown in Figure 1.

The scrubber was designed initially by examining the typical format of pathology reports from the three different institutions that participated. The scrubber was iteratively tested on small datasets, typically groups of 50–100 reports, until it could reliably remove common identifiers. Reports from all three institutions were used in this testing phase, but most of the reports used came from one institution, Department A.

The algorithmic structure of the scrubber involves three processes. First the program takes advantage of identifying information that may be present in the header of the file. The scrubber searches for any occurrences of these identifiers in the textual portions of the xml file (i.e. the body of the pathology report) and removes them. At this point the header information could also be completely removed, but in our environment, some of this information is required for later processing steps, so these fields are emptied just prior to the case being loaded into the SPIN network. The second step involves searching for predictable patterns likely to represent identifying data, such as dates, accession numbers, addresses, and proper names that can be found by way of markers such as Dr, MD, PhD etc. Additionally, names of institutions are identified by their common portions such as hospital, medical center, clinic, etc. These pattern searches are implemented as 50 "regular expressions" in Java [see Additional file 1] and stored in a separate file as variables. This modular construction allows easy modification of patterns or addition of new patterns to adapt the scrubber to new data sources with different conventions. As would be expected, the order in which the pattern searches are executed does play an important role since some expressions assume that certain patterns have already been removed.

Following this pattern matching step, the program then compares each word in the file to a database of personal and geographic place names. These names are stored in a MySQL database. The entries were derived from publicly available census lists of frequently occurring names from the 1990 census [17]. The lists contain over 90,000 unique first and last names, which represent about 90% of the last names encountered in the 1990 census. In addition, the US Census Bureau provides a gazetteer file [18] which contains US place names (cities, towns, etc). This file contains over 16,000 unique place names. These files were combined to form one table with over 101,000 unique entries. At one institution (Department A), this composite name table was augmented with the names of pathologists who were active during the period from which the reports were drawn. No institution-specific pathologist or patient names were added at the other two institutions.

A corpus of 1800 surgical pathology reports that had not been used previously for development or testing was extracted from the anatomic pathology laboratory information systems of three hospitals, with 600 cases being drawn from each institution. The reports were extracted in batches of 200 sequential case reports from three different time periods between 1999 to 2004. The reports from two hospitals included external consult cases, but in the third hospital these are separately accessioned with a unique set of numbers, so the extracted series contained no consultation reports. Since pathology departments do not usually retain tissue on external consult cases, there is little point in including consult reports in the SPIN network. The scrubber was therefore designed to segregate consult cases into a separate folder before removing the identifiers. Our analysis includes these consult reports, but when preparing reports for submission to the SPIN network we discard these consult reports.

Once the series of cases was extracted, custom programs unique to each institution were used to transform the raw extracts from the native laboratory information system format into XML files consistent with the schema used by the SPIN project. The SPIN schema allows for many different levels of detail to be encoded, but the major textual portions of the pathology report are stored intact in four elements (clinical information, gross description, diagnosis text and full text report). There are also separate elements for addendum text, electron microscopy findings, etc. Only surgical pathology case reports were used, but these reports included the results of special studies such as immunohistochemistry, flow cytometry, and immunofluorescence studies. No cytology or autopsy reports were included among the reports.

The scrubber software was installed on a personal computer running Microsoft Windows^® ^in each institution. The 600 individual XML files were processed by the scrubber in a batch. The scrubber searches through each text field and replaces any suspect words or text strings with a series of X's and a notation as to what type of information was presumably removed. The scrubbed file is saved under a new name so that the original file is not altered. In addition, the scrubber produces a log file with each character string that is removed from each file along with a reference to the reason that the string was removed (e.g. the type of the particular pattern match that led to the removal).

Following deidentification, the scrubbed files were manually reviewed by one of the pathologists (BB, UB, FK) in order to find and classify identifiers that were not removed by the scrubber. In addition, each original report was manually reviewed to count the total number of identifiers present in the text portions and to clarify any questions regarding whether a removed phrase was an identifier. The types of information that were counted as identifiers included the HIPAA enumerated list of identifiers (see Table 1) as well as the following information that is not required to be removed in a de-identified data set under HIPAA: all ages (not just those >89 years), all dates including year, states and countries, names of health care providers and health care organizations such as hospitals, medical laboratories, etc. An identifier was considered removed if a large enough portion of it was removed to make it very unlikely to be identifiable from the remaining fragment of text. For example, if "Jones Medical Associates" was present in the report and what remained after scrubbing was only "Medical Associates", this was considered to be a successful removal. Misspelled proper names (e.g. Smiht) were counted as identifiers since the correct spelling could usually be guessed (Smith). The logical portions of addresses (street number and name, city, state, country) were counted as separate identifiers for purposes of our analysis. Only the identifiers that were not removed by the software were classified as being a HIPAA identifier or non-HIPAA identifier.

## Results

**Figure 2:**
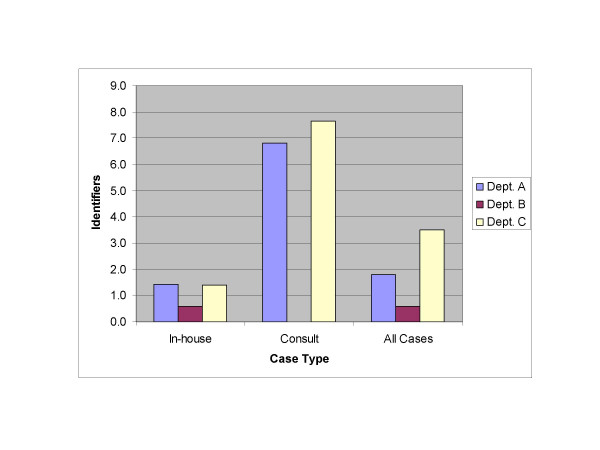
**Average number of unique identifiers present per report**. Note that Department B did not have any consult reports included in the sample.

The 1800 cases included 1559 (86.6%) in-house surgical pathology case reports and 241 (13.4%) external consultation case reports. 1254 (69.7%) reports contained at least one identifier. There were a total of 4515 identifiers present, including 3499 that were unique in a given report. The average number of identifiers was 2.5 per case, with 1.9 unique identifiers per case on average. The frequency of identifiers in reports varied markedly between type of case with all 241 (100%) external consults, but only 1013 of 1559 (65%) in-house case reports containing at least one identifier {p < 0.001 Chi-square}. The percentage of reports containing identifiers also varied between pathology departments with Departments A, B and C having 69.2%, 39.8% and 100% of cases with identifiers respectively {p < 0.001 Chi-square}. Additionally, the number of identifiers present in a report varied by both type of report and source institution (see Figure 2).

Since the presence of multiple instances of the same identifier in the same case report is unlikely to provide any additional information, the remainder of the analysis will focus solely on the identifiers that were unique in a given report. For example if the name "Jones" appeared twice in the text of three different reports in our testset, we ignored the second occurrence in each report and counted "Jones" as one identifier present in each report, for a total of three identifiers, not six. The deidentification software successfully removed the vast majority of identifiers. Of the 3499 unique identifiers present, 3439 (98.3%) were removed (see Table 2). Of the 60 identifiers that were not removed, 19 of them were HIPAA specified identifiers. Table 3 provides a breakdown of identifiers missed by type.

**Table 2:** Performance summary of the deidentification software

	Dept. A	Dept. B	Dept. C	Total
Reports	600	600	600	1800
Reports with any identifier	415	239	600	1254
Unique identifiers	1079	338	2082	3499
Unique identifiers per report	1.8	0.6	3.5	1.9
Unique identifiers removed	1057	320	2062	3439
Unique identifiers remaining, total	22	18	20	60
Unique HIPAA identifiers remaining	11	1	7	19
% Unique identifiers removed	98.0%	94.7%	99.0%	98.3%
				
Unique over-scrubs	1126	961	2584	4671
Unique over-scrubs per report	1.9	1.6	4.3	2.6
% unique phrases removed that were identifiers	48.4%	25.0%	44.4%	42.4%

**Table 3:** Summary of identifiers that were not removed.

Identifier	Identifier Type	In-house Cases	Consult Cases	Total
Accession number	HIPAA	0	10	10
Patient Name				
Misspelled	HIPAA	5	2	7
Correctly spelled	HIPAA	0	0	0
Medical record number	HIPAA	1	0	1
Date	HIPAA	1	0	1
HIPAA subtotal		7	12	19
				
Institution address, partial	Non-HIPAA	0	17	17
Age <90	Non-HIPAA	16	0	16
Health care organization name	Non-HIPAA	0	6	6
Doctor name	Non-HIPAA	1	1	2
Non-HIPAA subtotal		17	24	41
				
Grand total	HIPAA and Non-HIPAA	24	36	60

There were a total of 3499 unique identifiers in the test reports, of which 1809 were from consult reports and 1690 from in-house reports.

Comparing performance by source institution, the percentage of unique identifiers removed varied between 94.7 and 99% (see Table 2). This performance was statistically different {p < 0.001 Chi-square}. Another way to look at the data is to consider in-house and external consult cases separately. The 241 consult cases contained slightly more than half of the identifiers (1809 of 3499, 51.7%). We can see in Table 3 that the majority, 36 of 60 (60%), of missed identifiers were in consult case reports, which is not surprising since these types of reports often contain listings of slide labels, accession numbers, outside report numbers, and patient identifiers which are directly dictated into the text of the reports. They also contained the majority of HIPAA specified identifiers that were missed, 12 of 19 (63%).

Names are one of the identifiers of greatest concern since these provide a fairly high level of identification on their own without reference to any other source of information. Fortunately, in many institutions patient names are only rarely included in the body of the report. However, some institutions have gross dictation policies that include documenting the name that is present on specimen containers (e.g. "Received in formalin labeled 'Mary White, left fallopian tube' is a piece of pink tan soft tissue..."). When the name is spelled correctly and it matches either a name in the header information of the report or one of the words in the proper name database, it can be successfully removed. However, when the name is misspelled, we must rely upon other clues, such as inclusion in quotation marks, capitalization, proximity to a correctly spelled proper name, or proximity to a marker word such as "labeled". As evident in Table 3, all correctly spelled names were removed, but seven misspelled names were not removed. Four of these names were first names and three were last names. All seven misspelled names came from the reports of one hospital, indicating that this is an issue related to the style of reporting at that institution.

Numbers are another source of concern since some types of numbers can be uniquely identifying when cross referenced, such as Social Security numbers or medical record numbers. Many such numeric identifiers are easily dealt with since the length and form may be known in advance (e.g. Social Security numbers, zip codes and phone numbers). Medical record numbers can usually be easily removed since they tend to be similar from institution to institution. In this trial, there was one medical record number missed from an in-house case. The number was missed because it had an unusual spacing pattern dividing the seven digit number into three groups of two and three digit numbers. This particular type of error should be easy to rectify by altering the pattern matching algorithm to take account of common spacing characters such as dash, period, space, slash, backslash, etc.

Accession numbers provide a more complicated problem than medical record numbers. In our sample, there were 10 accession numbers that were not removed, all of which were contained in outside consultation reports. Accession numbers are a type of information that is specifically enumerated by HIPAA, but they likely represent a lower level of risk for re-identification since one would need to know which institution an accession number was from and have access to their records in order to attempt re-identification. None of these reports where an accession number was missed by the software had any additional information relating to the institution that was not removed. The reason that pathology case accession numbers are challenging to remove is the wide variety of formats being used. Even within a single institution there may be multiple different formats. These formats often include letters which denote whether a sample is for cytology, surgical pathology or hematopathology sample. Slide numbers are often a special case of this class of identifiers where there is an extension added, such as "S05-12345A B1-L2". While the scrubber can be easily tailored to remove accession numbers that are in the specific format used in a particular institution, consult cases provide seemingly endless examples of ingenious variations on these patterns. One particularly problematic issue is that short accession numbers may be identical to medically important terms, such as "CD-34". Most surgical pathology departments process thousands of samples a year, so most accession numbers contain 4 or more digits. However many dictators and transcriptionists do not include leading zeros, so specimens with low accession numbers may be especially problematic.

Dates and ages are another type of numeric data that can generally be removed successfully. Our software missed only one date (month/year format) in our test set but missed 16 ages, 15 of them because of the convention in one institution of denoting ages by the abbreviation "y.o." (meaning "years old"). The scrubber was updated to take this into account and subsequently removed all such ages in the test set. However ages are sometimes free standing numbers in the clinical history section of pathology reports (e.g. "50, screening colonoscopy"). In this case the only way to identify these ages is to depend on location and contextual clues. The final age that was missed by our scrubber was in the form "three and one-half year old," which points out that ages which are fractional and/or written out in words must be considered as well. While HIPAA allows ages less than 90 years to be included in de-identified reports, we feel that it is safer to exclude all ages from the text of pathology reports if possible. One approach to the issue of numeric identifiers is completely removing all the numbers from a report. This should completely remove or obscure numeric identifiers such as accession number, medical record number, etc. The problem with this approach is that it removes many important numbers such as tumor size, number of positive lymph nodes, distance to uninvolved margin, tumor grade and so on.

The remaining types of identifiers that were missed by our deidentification software were the names of health care organizations (hospitals, pathology departments, etc.), partial addresses of such institutions and a single name of a physician involved in the patient's care. Once again, these are not identifiers that are mentioned by HIPAA, but we have attempted to remove them in order to provide the most stringent deidentification possible. This is particularly important with regard to consult cases, since as we have already noted, external accession numbers are difficult to remove with high confidence and the combination of an accession number and the name of the health care organization which generated it, provides a higher likelihood of re-identification, at least theoretically. Our current implementation of the scrubber is limited by the fact that we only included a place names list of U.S. locations and one of the institutions that supplied reports has an appreciable number of international consultations. Not surprisingly, our scrubber did not fare well at removing these foreign location names. This is another example of where tailoring the scrubber to the particular content of reports will be very valuable.

We also evaluated the performance of our scrubber with regard to incorrectly removing words or phrases that did not contain identifiers. The number of unique "over-scrubs" was 4671, giving an average of 2.6 phrases incorrectly removed per report (see Table 2). The amount of over-scrubbing ranged from 1.6 to 4.3 per case. The ratio of removed phrases that contained at least one identifier to the total number of removed phrases was 42.8%. The over-scrubbing appears to be primarily related to the large number of words that are contained in our proper names and place names table. We manually removed a few words from our name list if they gave vast excesses of over-scrubbing as compared to valid identifier removal. Some of these problematic words remained during our test, though. For example, one institution routinely used the word "toto" – this single word was responsible for 387 instances of over-scrubbing. Other words that were commonly over-scrubbed include color names such as "black" and "orange" and anatomic locations such as "L4-5" and "R4" used in spinal procedures and prostate biopsies respectively. Any deidentification system will need to be tuned to adjust the specificity of the phrase removal to the desired level. In our case, the average of 2.6 over-scrubbed phrases per case was fairly low and the pathologists reviewing the scrubbed reports felt that the reports were still easily understandable with information crucial for understanding the report contents being removed incorrectly only rarely.

The speed of the scrubber was also evaluated by running a set of 6600 cases from one of the institutions. Deidentification took 140 minutes, which works out to an average speed of 47 cases per minute. It would be expected that the speed of the deidentification would vary with the amount of text in each report, but the speed of the software is sufficiently fast that a large number of cases can be processed quickly. At this speed, over 65,000 cases of typical length can be processed per day, making it suitable for high volume applications.

## Discussion

The primary concern regarding deidentification software such as this is how well it performs at removing identifiers and leaving behind the remainder of the text. The software described in this paper performs well, but not perfectly in this regard, removing 98.3% of unique identifiers in our test set. Notably, the scrubber performance varied by institution, ranging from 94.7% to 99.0% removal of identifiers. This performance appears to be similar to the few other comparable studies available. Gupta et. al [4] reported on a multi-step trial of a deidentification software engine and by the third round, only 8 reports out of 300 had organizational or personal names or accession numbers missed. Thomas et. al. [5] developed a tool to remove names in pathology reports based on pairwise occurrence and presence of marker words such as Mr., Dr. etc. Their software found 228 of 231 names (98.7%).

Our report is unique in that the number of identifiers was quantified and our data is reported as a percent of the number of identifiers as opposed to a percent of reports containing missed identifiers. Our data also highlights the wide variance in number of identifiers between external consult and in-house cases as well as between different institutions. The number of identifiers per report varied almost 6-fold in our sample, ranging from 0.6 to 3.5. Somewhat surprisingly, the scrubber performed best in this trial on the reports that contained the most identifiers. One reason for this may be that the scrubber was trained primarily on reports from Department A, which contained many identifiers. The reports chosen for the trial from Department B, while having a low number of identifiers, did use one age convention that we had not anticipated. When a simple change was made to the scrubber, 15 of the 18 identifiers that were missed in the trial were now removed, which gives a percentage of removed identifiers of 99.1%, which is comparable to the performance on the reports from Departments A and C.

A somewhat different approach that has been suggested [7] for deidentification of surgical pathology and other textual documents which involves autocoding phrases that are contained in a reference terminology (e.g. UMLS), retaining high frequency "stop" words and discarding the remaining words. Any terms that are found in the reference terminology are then transformed by substituting synonyms in place of the original text. This results in a document that arguably contains all the same concepts in the same order, but which should be bereft of all identifiers. We agree that this should provide a high level of deidentification, but it does have a few limitations, which are discussed by the author. In our opinion, the major limitation of this procedure is that the output is difficult for a human to read, especially when obscure synonyms are used, and some of the meaning will be lost in cases where crucial words or phrases are not present in the reference terminology.

Our results corroborate the observations of prior authors that it is extremely difficult, if not impossible, to automatically remove all possible identifiers from medical text while leaving the remainder intact. The difficulties include misspelling, which may be particularly challenging to detect, especially if the misspelling results in a valid word or even worse a valid word with a specific medical meaning. Use of an automated spell-checker prior to scrubbing could be tested to see if it improves identifier removal. Additionally, medical eponyms are particularly challenging, as are names that have other common meanings (e.g. "Black" and "Brown" are common surnames, but often occur in gross descriptions of tissue and may not be reliably distinguished by capitalization). Another possible issue is that patients with rare diseases may be identifiable by the disease name and general geographic area. For example, progeria is a very rare condition associated with unnaturally fast aging. It is estimated that there are less than twenty people in the United States currently living with this disease, so simply knowing that a report concerns a patient with this condition makes it likely that an exact identification could be made.

One of the few benefits of the perennial problem of receiving specimens with little in the way of clinical information is that the clinical information section of a pathology report is usually the place where unusual social or clinical information would be located, such as circumstances of traumatic injuries which might be publicly known, for example victims of plane crashes or suspected terrorist incidents. Indirect identifying information such as "head researcher at local genetics firm" are notoriously difficult to find and remove since they do not contain specific identifiers, but can effectively limit the number of possible individuals greatly. These types of identifiers probably fall under the final category of HIPAA-specified identifiers, "any other unique identifying number, characteristic, or code" [2]. None of the reports used in our test were felt to contain this type of identifier (judged by pathologist reviewing the cases), but this remains a potential issue with any sort of scrubbing procedure based on pattern matching alone.

While our results are encouraging, there is clearly room for improvement in our tool. The major areas where improvements are needed include better ways for locating and removing misspelled names, outside accession numbers, and addresses. The inclusion of foreign addresses is particularly challenging, since many of the words in such addresses will not be found in English language lists and there are a variety of different address formats in use throughout the world. This points out the need for continued monitoring and improvement of any scrubbing tool, especially when expanding the types or sources of reports to be scrubbed. In our tests, we noted changes in report styles within an institution over time. The introduction of new terms or abbreviations which may be mistaken for identifiers (e.g. Her-2) may also require subsequent revalidation. While ongoing quality assurance is a time consuming process it is extremely valuable and highly recommended. Finally, changes made to the algorithms may inadvertently cause unanticipated problems and therefore a set of reports that can be used repeatedly as a quality control measure would be highly desirable, as suggested by Gupta [4]. We have the collection of original reports used in this study and we have subsequently run later versions of the scrubber over these reports, but we have not yet developed an automated tool for evaluating the results. We have presented our work to five Institutional Review Boards and each has approved the use of this tool for the purpose of deidentifying pathology reports for inclusion in the SPIN demonstration network as well as our local version, the Virtual Specimen Locator of the Dana Farber Harvard Cancer Center. We have scrubbed over 200,000 cases with satisfactory results to date. However, it must be kept in mind that deidentification processes such as ours do have limitations and it is safest if de-identified reports are restricted to use by investigators who have signed data use agreements that include prohibitions on attempting to re-identify the subjects of such reports. In such a setting, the risk to patients and research subjects is low and is consistent with the statutory protections described in HIPAA and the Common Rule.

## Conclusion

Deidentification of medical data is likely to increase in importance as the volume of electronic medical records grows. The need for reliable tools to deidentify such data has been recognized, but freely available tools are rare. This work demonstrates that an open-source tool can be built which performs well in the domain of free-text pathology reports and emphasizes the challenges inherent in such reports. Due to the wide variance in report language and styles, a system like this will perform well only if tailored to a particular type of report and institutional style. Our software can provide a basis for others to develop robust and capable tools for deidentifying a wide variety of medical text.

## Availability and requirements

Project Name: Shared Pathology Informatics Network

Project Home Page: 

Operating System: Platform independent

Programming Language: Java

Other Requirements: JDK 1.4.2, JDOM beta 9, MySQL 4.0X, MySQL ODBC Bridge Driver

License: GNU GPL

Any restrictions to use by non-academics: None

## Competing interests

The author(s) declare that they have no competing interests.

## Authors' contributions

BB contributed to the algorithmic design of the scrubber software, performed the initial validation and iterative testing, designed the validation study, participated in data collection and analysis and wrote the manuscript. RM contributed to the algorithmic design and coded all of the software, participated in the initial validation and iterative testing and participated in data collection. UB contributed to the algorithmic design of the scrubber software and participated in the data collection. FK contributed to the algorithmic design of the scrubber software, participated in the initial validation and iterative testing and participated in the data collection and analysis. All authors read and approved the final manuscript.

## Pre-publication history

The pre-publication history for this paper can be accessed here:



## Supplementary Material

Additional File 2 HMS Scrubber version 1.0 beta distribution files. This archive contains all of the java code and supporting files needed to install and run the scrubber (provided that the needed versions of Java, MySQL and JDOM are present). Click here for file

Additional File 1 This is a text file, which contains the java code with the approximately 50 regular expressions used by the scrubber software. It is provided as a separate text file as a convenience. Click here for file
