# Supplementary material for: Development and evaluation of an open source software tool for deidentification of pathology reports
Source: BMC Med Inform Decis Mak. 2006 Mar 6;6:12. doi: 10.1186/1472-6947-6-12 (PMC1421388; doi:10.1186/1472-6947-6-12)
Supplement: Additional File 2 — HMS Scrubber version 1.0 beta distribution files. This archive contains all of the java code and supporting files needed to install and run the scrubber (provided that the needed versions of Java, MySQL and JDOM are present). [file 1472-6947-6-12-S2.zip › docs/ScrubberReleaseNotes.doc]

Documentation for the HMS Pathology Report SCRUBBER

Version: 1.0 beta
Date: 12/7/2005

Disclaimer:
This is a program designed to remove identifying information from pathology
reports which have been into valid SPIN XML format files. This version is a beta version and will NOT always remove all identifiers from a file. Significant knowledge of Java, Regular Expressions and MySQL as well as the characteristics of your input files will probably be needed to make this code function acceptably. No warranties as to the performance of this code in any situation are given or implied. The suitability of the program's performance MUST be established by the user.  

Updates: 
This software is continually being updated and modified.  
For updates please check the SPIN Website, 
http://spin.nci.nih.gov

Copyright:  
This software is copyright 2005 by Harvard Medical School.  

License Terms:
This file is part of the HMS Pathology Report Scrubber.

The HMS Pathology Report is free software; you can redistribute it and/or modify it under the terms of the GNU General Public License as published by the Free Software Foundation; either version 2 of the License, or  (at your option) any later version.

The HMS Pathology Report Scrubber is distributed in the hope that it will be useful,
but WITHOUT ANY WARRANTY; without even the implied warranty of
MERCHANTABILITY or FITNESS FOR A PARTICULAR PURPOSE.  
See the GNU General Public License for more details.

You should have received a copy of the GNU General Public License along with this program; if not, write to the Free Software Foundation, Inc., 59 Temple Place, Suite 330, Boston, MA  02111-1307 USA or check the GNU website at http://www.gnu.org/licenses/gpl.html

Software Requirements:
1	Windows (any version)
2	JDK 1.4.2 			http://java.sun.com/j2se/1.4.2/
3	MySQL Database 		http://www.mysql.com/
CHIRPS compliant XML 	Schema is included in this software distribution

Installing the Scrubber database:

1)	Download and Install MySQL 4.0X 

Create the scrubber database using the provided SQL script. 
	
	For example:
	mysql> source C:\HMS_Scrubber_v1.0b\DatabaseFiles\createdb.sql

Copy the Names and States 

	3.1 Shutdown Mysql 

	For Example:
	C:\> net stop mysql 

	3.2 Copy the Names and States to the Scrubber database

	For Example:
	C:\>copy C:\HMS_Scrubber_v1.0b\DatabaseFiles\names* C:\mysql\data\scrubber
      C:\>copy C:\HMS_Scrubber_v1.0b\DatabaseFiles\states* C:\mysql\data\scrubber

4)  Start Mysql

	For Example:
	C:\> net start mysql 

Preparing the Scrubber Input:
Before running the Scrubber class, the input data must be transformed into individual XML files which are compliant with the SPIN schema.  Each pathology report must be contained within a single XML file.

Running the Scrubber Program:

Example Usage: 
1	Scrubber.bat 
2	Scrubber.bat  C:\HMS_Scrubber_v1.0b\TestCase\
3	Scrubber.bat  C:\\HMS_Scrubber_v1.0b\TestCase\ 	C:\xmloutput\

Defaults:
1	inputDirectory    =  c:\BWH
2	outputDirectory  =  directory named “output” under the input directory 

Note:
All the words that are replaced are written to the “replacedtext.txt file” which is created under the temp directory under the specified input directory .

Testing that the scrubber is working:

In a folder called “TestCase” there is a valid SPIN XML file which contains a made up pathology report with invented patient information named “testcase.xml” that can be used to verify that the scrubber is functioning at a basic level.  The files “scrubbedtestcase.xml” and “Replaced Text for testcase.txt” can be compared with the output of your installation of the scrubber.  The scrubber should remove the name “John Doe” from the pathology report file.

Extending Scrubber Functionality:

The names and states can be dynamically extended by adding entries to the NAMES and STATES tables of the scrubber database. For instance, a list of patient and doctor names can be loaded in the names database to supplement the current people names listing (which was derived from US census files).

If new regular expressions (regex) need to be added, they can be defined in the RegexStrings class.  The “TestRegex” class needs to be changed to include code to create a pattern for the newly added regex so that it can be found and replaced in the input string. Also, this is where code is added to write the replaced strings in the replacedtext.txt file.

Revision History:
Version 0.1 alpha released 9/16/03 – Initial release for testing and feedback 

Version 1.0 beta released 12/1/2005 – Second release which updates the structure of the database tables, includes various bug fixes and simplifications for users.  Also, the scrubber attempts to identify and segregate pathology report files which represent outside consultation cases.
